# Left Atrial Myxoma Presenting as Lateral Medullary (Wallenberg's) Syndrome

**DOI:** 10.1155/2019/5610213

**Published:** 2019-11-11

**Authors:** Simran Gupta, Ricky Ayala, Aakash Desai, Viraj I. Modi, Robert J. Nardino

**Affiliations:** Department of Internal Medicine, University of Connecticut School of Medicine, UCONN Health, Farmington, CT, USA

## Abstract

Myxomas are benign, primary tumors of the heart. Atrial myxomas can present with a variety of clinical features including dyspnea, orthopnea, pulmonary edema, and pulmonary or systemic emboli. Constitutional symptoms such as fever and weight loss may also be present. We report the case of a young female presenting with headache, facial numbness, and vertigo, who was found to have a posterolateral medullary stroke secondary to a large left atrial cardiac myxoma.

## 1. Learning Objective

Cardioembolic origin is a known cause of ischemic stroke. Differential diagnoses most often include atrial fibrillation and patent foramen ovale. Atrial myxoma is a rare and potentially dangerous cause of ischemic stroke and should be considered on a differential for cardiac etiology.

## 2. Introduction

The most prevalent primary cardiac tumors, myxomas, most often grow in the atria. About 75% are located in the left atrium and 18% in the right atrium (1, 2). Classically they grow at the fossa ovalis border of the interatrial septum (3). Myxomas most commonly occur in middle age, though rare occurrences in the pediatric population have been reported (1, 3). They are more predominant in women than men (1, 4). Previous studies have shown that patients with myxomas can have a variety of initial presentations, including obstructive symptoms (67%) and embolic symptoms (29%) (5). Other studies have focused on symptoms based on location of the tumor, with septal tumor location strongly associated with congestive heart failure and extraseptal tumors associated with neurological events (6). Most commonly though, most studies conclude that myxoma symptoms can mimic many cardiovascular diseases, and thus a high index of suspicion is important (7). We report the case of young female who presented with a headache, facial numbness, and vertigo who was found to have a posterolateral medullary stroke which was secondary to a large left atrial cardiac myxoma.

## 3. Case Presentation

**Figure 1:**
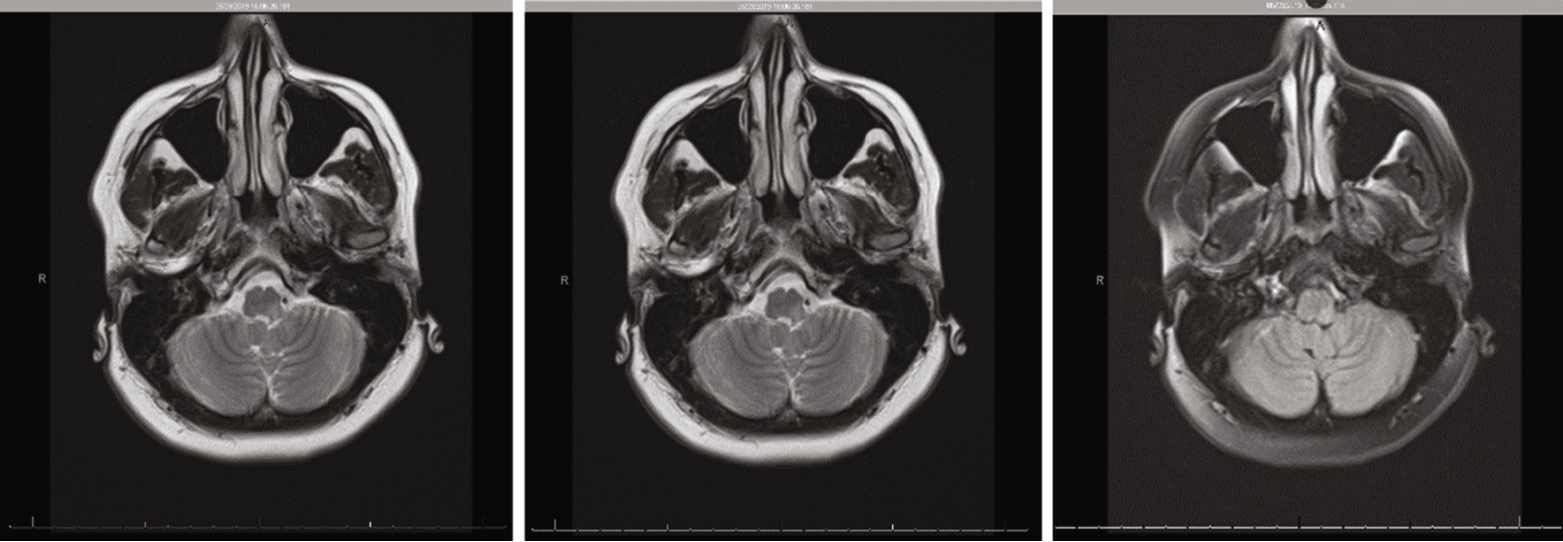
MRI of the brain with and without contrast showing focal restricted diffusion within the left posterolateral aspect of the medulla with associated focal heterogeneous T2 hyperintense signal, indicative of an evolving focal subacute cerebrovascular accident. Alternatively, this finding may represent an acute-subacute focus of demyelination in the proper clinical setting.

A 48-year-old female with a history of hypertension, migraines, anxiety, and depression presented to the Emergency Department with a left-sided occipital headache for one hour (thought to be consistent with her usual migraine presentation) with accompanied numbness of the left face and lips, vertigo, and lightheadedness. Her initial blood pressure was 172/85, and cranial nerves II through XII were intact except for some mildly decreased sensation to light touch on the left side of her face. The patient did note that she felt less vibration from the tuning fork on the left forehead as compared to the right forehead. Cognitive status and language were grossly intact and muscle strength was 5/5 in all extremities. Initial NIH stroke scale was 1. On cardiac examination, the patient had an extra diastolic heart sound, interpreted as a splitting of S2, without murmurs, gallops, or rubs. Given concern for a stroke, an initial computed tomography (CT) scan of the head was performed and was found to be negative for an acute, evolving cerebrovascular accident. It was thought that the symptoms were most likely related to a migraine, but the patient was admitted to the floors for further workup. Over the next 24 hours, the patient's clinical condition continued to deteriorate with a worsened neurological exam now including diplopia, left-sided dysmetria, more pronounced vertigo, and difficulty swallowing. A stroke alert was called and magnetic resonance imaging (MRI) of the brain was performed, which showed focal restricted diffusion within the left posterolateral aspect of the medulla with associated focal heterogeneous hyperintense signal, indicative of an evolving focal subacute cerebrovascular accident (Figure 1). The patient was transported to the step-down unit with an NIH stroke scale score of 2 for neurological checks every two hours. It was noted that the patient was not a candidate for tPA at this time due to presence of symptoms ongoing for over 24 hours.

**Figure 2:**
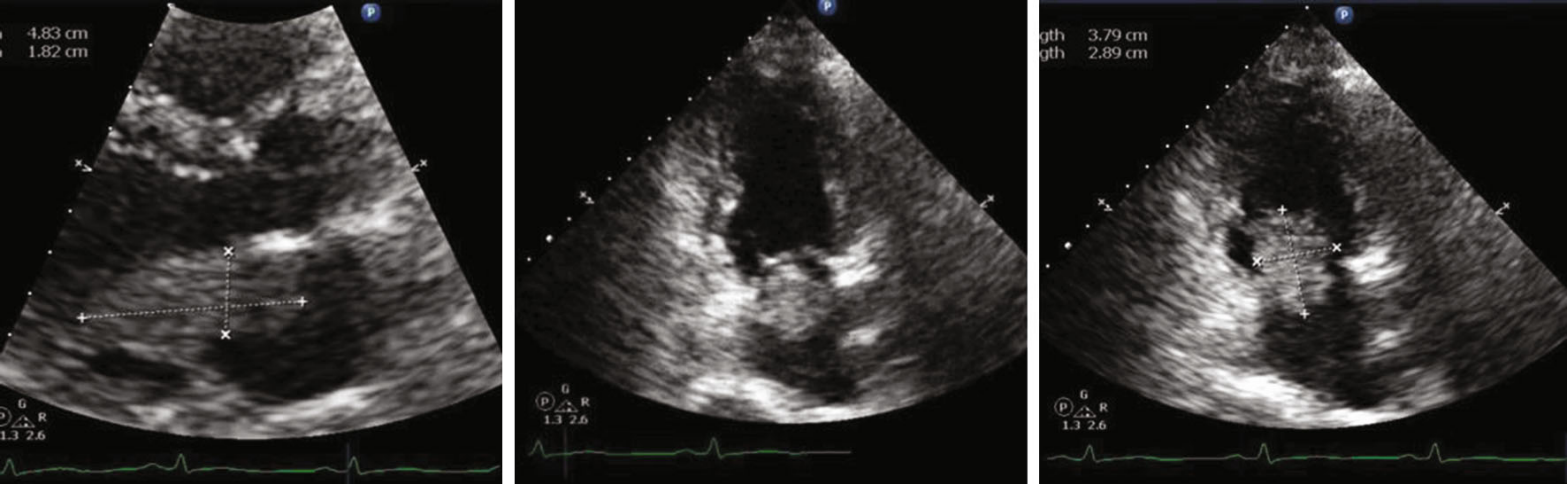
Transthoracic echocardiogram. There is a large, 5.7 *cm* (*L*) × 1.9 *cm* (*W*), mobile mass on the left side of the interatrial septum; the appearance is consistent with myxoma (echocardiographic contrast reveals vascularity of the mass). The mass travels along with the anterior mitral leaflet and plops in and out of the left ventricle. Transvalvular velocity of the mitral valve is within the normal range, and there is no evidence of stenosis. Left ventricular ejection fraction was in the range of 55% to 60%.

Given her presentation and imaging findings, she underwent further workup including a transthoracic echocardiogram (TTE). TTE demonstrated a large, 5.7 cm × 1.9 cm, mobile mass on the left side of the interatrial septum consistent with a myxoma (echocardiographic contrast reveals vascularity of the mass). The mass traveled along the anterior mitral leaflet and plopped in and out of the left ventricle (Figure 2). The patient's left ventricular ejection fraction was preserved, and there was no wall motion abnormality. Based on location and size of the mass, there was serious concern for complete obstruction of blood flow through the heart. Further history elicited from the patient at this time revealed that she had a long-standing history of dizziness and lightheadedness.

Cardiothoracic surgery was consulted and they took the patient to the operating room within 12 hours of echocardiography. The surgeons used a median sternotomy technique to enter the chest cavity, the aorta was cross clamped, and the heart was arrested with 1 liter of antegrade first warm then cold hyperkalemic blood cardioplegia. The right atrium was entered and the incision was carried cephalad toward the interatrial septum. The roof of the left atrium was then incised and the incision was carried toward the fossa ovalis, at which point the tumor was carefully excised off the septum. The incisions were closed carefully and the heart was deaired and the aortic cross clamp was removed. The heart did not require defibrillation and spontaneously returned to normal sinus rhythm. Upon reaching normothermia, the patient was easily weaned from cardiopulmonary bypass on no pressors or ionotropes. The patient tolerated the procedure well and was monitored in the intensive care unit for 24 hours and then transferred to a monitored step-down unit. Her stroke symptoms continued to persist through the postoperative stay, with some resolution of diplopia. She was discharged after two days in step-down to a long-term acute care facility for extensive rehabilitation of her stroke symptoms. A repeat transthoracic echocardiogram obtained one month after surgery showed no evidence of myxoma recurrence, with a normal left ventricular ejection fraction.

## 4. Discussion

Stroke, or central nervous system infarction, is broadly defined as brain, spinal cord, or retinal cell death (8). Ischemic stroke in particular is defined as “an episode of neurologic dysfunction caused by focal cerebral, spinal, or retinal dysfunction.” Cardioembolic origin accounts for approximately 14-30% of ischemic strokes, with atrial fibrillation being the most common (45%) underlying condition (9). Other common etiologies include infective endocarditis, valvular heart disease, and much more rarely, cardiac myxoma. Previous research has shown that cardiac myxomas account for only approximately 0.5% of strokes (9, 10). The classic triad of symptoms of cardiac myxomas includes obstructive, embolic, and constitutional manifestations (2). Obstructive symptoms, which are the most common initial symptoms, include dyspnea, cough, and heart failure (4). Embolic symptoms usually present as peripheral, pulmonic, or cerebral. Constitutional symptoms are thought to be driven by tumor cell release of IL-6 and include arthralgias, fatigue, weight loss, fever, and Raynaud's phenomenon (5). Patients often have some or all of these symptoms on initial presentation, and severity depends on the tumor size, location, and mobility. Our patient, with a large obstructing left atrial myxoma accompanied by lightheadedness and stroke, displayed obstructive and embolic symptoms.

Lateral medullary, or Wallenberg's, syndrome is defined as a neurological condition due to a stroke in the vertebral or posterior inferior cerebellar artery of the brain stem (11). Symptoms may include difficulty of swallowing, dizziness, nausea and vomiting, decreased pain and temperature sensation on one side of the face and opposite side of the body, and problems with coordination. During her hospital course, our patient noted almost all of the above symptoms. Treatment is predominantly symptomatic, and prognosis is dependent on size and location of the affected brain stem area. To our knowledge, this is only the second reported case of Wallenberg's syndrome as the initial presentation in a patient with a left atrial myxoma. Cerebral vascular accidents in general are a known complication, however, of atrial myxomas, particularly those of the left atrium. Regardless of presenting symptoms, research has shown that it is important to consider myxoma on the differential diagnosis of stroke origin. The American Heart Association guidelines propose that initial evaluation of an embolic event should include echocardiography to investigate intracardiac causes (12). A study of 24 patients with cardiac myxomas in Taiwan also suggests that echocardiography is the most useful diagnostic tool for cardiac myxomas and serial echocardiograms (every six to twelve months) may be needed after resection to monitor recurrence. (7).

Siminelakis et al. showed that urgent surgical resection via median sternotomy with the patient on cardiopulmonary bypass under mild hypothermia is the treatment of choice (2). Using a technique described by Siminelakis et al. (2), they suggest the ideal approach to a left atrial myxoma occupying the majority of the left atrium is an incision starting from the pulmonary vein continuing vertically to the interatrial septum and the free wall of the right atrium. This would allow complete visualization and opening of the left atrium. As described above, this was the surgical technique employed by the cardiothoracic surgeons for our patient. Other studies have shown that the ideal surgical technique is right atriotomy with excision of the fossa ovalis and surrounding tissues and closure with a pericardial patch (2). Regardless of technique, it is important to achieve complete resection of the tumor with a margin of safety to minimize chance of recurrence (13). With complete resection of the tumor, the risk of recurrence is approximately 3% (14, 15). Regular and long-term follow-up with clinical examinations and serial echocardiography is highly recommended (16), though a consensus on total duration of follow-up time has not been established.

## 5. Conclusion

This case highlights the variety of presentations that can be associated with cardiac myxomas and emphasizes the need for obtaining an echocardiogram as well as considering cardiac myxoma as a possible differential diagnosis for stroke. Our patient was a young, relatively healthy female with few risk factors for stroke. However, she did have a long-standing history of dizziness, a symptom that could be explained by the tumor. Given the size of her myxoma, a delay in diagnosis could have been fatal. In a patient without major risk factors, who has an unusual or unclear presentation, it is imperative to consider myxoma as a part of cardiac etiologies and intervene early.
